# Landscape and future directions of machine learning applications in closed-loop brain stimulation

**DOI:** 10.1038/s41746-023-00779-x

**Published:** 2023-04-27

**Authors:** Anirudha S. Chandrabhatla, I. Jonathan Pomeraniec, Taylor M. Horgan, Elizabeth K. Wat, Alexander Ksendzovsky

**Affiliations:** 1 School of Medicine, University of Virginia Health Sciences Center, Charlottesville, VA 22903 USA; 2 Surgical Neurology Branch, National Institutes of Neurological Disorders and Stroke, National Institutes of Health, Bethesda, MD 20892 USA; 3 Department of Neurosurgery, University of Virginia Health Sciences Center, Charlottesville, VA 22903 USA; 4 Department of Neurosurgery, University of Maryland Medical System, Baltimore, MD 21201 USA

**Keywords:** Neurosurgery, Movement disorders, Therapeutics

## Abstract

Brain stimulation (BStim) encompasses multiple modalities (e.g., deep brain stimulation, responsive neurostimulation) that utilize electrodes implanted in deep brain structures to treat neurological disorders. Currently, BStim is primarily used to treat movement disorders such as Parkinson’s, though indications are expanding to include neuropsychiatric disorders like depression and schizophrenia. Traditional BStim systems are “open-loop” and deliver constant electrical stimulation based on manually-determined parameters. Advancements in BStim have enabled development of “closed-loop” systems that analyze neural biomarkers (e.g., local field potentials in the sub-thalamic nucleus) and adjust electrical modulation in a dynamic, patient-specific, and energy efficient manner. These closed-loop systems enable real-time, context-specific stimulation adjustment to reduce symptom burden. Machine learning (ML) has emerged as a vital component in designing these closed-loop systems as ML models can predict / identify presence of disease symptoms based on neural activity and adaptively learn to modulate stimulation. We queried the US National Library of Medicine PubMed database to understand the role of ML in developing closed-loop BStim systems to treat epilepsy, movement disorders, and neuropsychiatric disorders. Both neural and non-neural network ML algorithms have successfully been leveraged to create closed-loop systems that perform comparably to open-loop systems. For disorders in which the underlying neural pathophysiology is relatively well understood (e.g., Parkinson’s, essential tremor), most work has involved refining ML models that can classify neural signals as aberrant or normal. The same is seen for epilepsy, where most current research has focused on identifying optimal ML model design and integrating closed-loop systems into existing devices. For neuropsychiatric disorders, where the underlying pathologic neural circuitry is still being investigated, research is focused on identifying biomarkers (e.g., local field potentials from brain nuclei) that ML models can use to identify onset of symptoms and stratify severity of disease.

## Introduction

Brain Stimulation (BStim) is a surgical technique that uses implantable electrodes in deep brain structures to modulate aberrant neural circuits^1^. Leveraging electricity for brain lesioning began close to a century ago, though neuromodulation to address specific neurological diseases only started gaining traction in the 1970s. Almost 30 years later, in 1997, the Food and Drug Administration (FDA) approved BStim for the “…suppression of tremor due to essential tremor or Parkinson’s disease; unilateral or bilateral”^2^.

The breadth of indications for BStim has increased over the past 15–20 years. While BStim is still predominately employed for movement disorders such as Parkinson’s disease (PD) and essential tremor (ET), novel techniques and brain targets are enabling broader clinical indications. Increased understanding of aberrant brain circuitry in neuropsychiatric diseases, such as depression and compulsive disorders, also brings new or improved targets, such as the anterior limb of the internal capsule, sub-thalamic nucleus, globus pallidus internus, and nucleus accumbens (Table 1)^3^.

**Table 1:** Potential targets for BStim by indication. Most indications have BStim targets across thalamic, sub-thalamic, and non-thalamic locations. Most work to date has involved thalamic and sub-thalamic targets.

Indication	Subset of potential targets for *BStim*		
	Thalamic	Sub-thalamic	Non-thalamic
Epilepsy^1^	Anterior nucleus Centromedian nucleus	Subthalamic nucleus	Amygdala Hippocampus Cerebellar hemisphere
Essential tremor^2,3^	Ventral intermediate nucleus Subthalamic nucleus	Posterior subthalamic area Zona incerta	–
Parkinson’s disease^4^	Ventralis intermedius nucleus	Subthalamic nucleus	Pedunculopontine nucleus Globus pallidus internus
Tourette’s^5,6^	Centromedian-parafascicular complex	–	Ant. limb of internal capsule Globus pallidus internus Nucleus accumbens
Depression^7,8^	–	Inferior thalamic peduncle	Anterior cingulate cortex Nucleus accumbens Ventral capsule/ventral striatum
Schizophrenia^9,10^	–	–	Substantia nigra pars reticulata Anterior cingulate cortex Nucleus accumbens Ventral capsule/ventral striatum
Obsessive-compulsive disorder^11,12^	–	Subthalamic nucleus	Ant. limb of internal capsule Internal capsule nucleus accumbens Ventral capsule/ventral striatum

Most BStim systems are “open-loop” and deliver constant stimulation based on manual parameter adjustment^4^. This technology is slowly starting to evolve into “closed-loop” systems that analyze neural biomarkers (e.g., local field potentials) to then automatically adjust modulation parameters in a dynamic and patient-centric manner. Designing closed-loop systems present unique challenges of understanding, in real-time or near real-time, individual brain states and contexts to calculate and deliver precise stimuli that return patients to a more functional baseline.

Due to increased reliance of closed-loop systems on larger idiosyncratic datasets, significant research is being conducted to apply machine learning (ML) methods to tailor BStim systems to individual patients, thereby increasing safety and efficacy. The current review aims to summarize the current state of ML techniques applied for the development of closed-loop BStim systems to treat epilepsy, movement disorders, and neuropsychiatric disorders.

## Review of literature

### Epilepsy

Epilepsy is a relatively common neurological condition^5,6^ that results in recurrent seizures. Unfortunately, initial treatment with oral antiseizure medications is only effective for approximately 50% of patients^7–9^. Failure of first-line medications is managed with second-line medication or combination therapy^10^. On top of drug-related side effects (e.g., nausea, vomiting, dizziness, tremor, confusion, and drowsiness), roughly 30–40% of patients with epilepsy do not adequately respond to drug therapy. In those patients, resective surgery is an option that can sometimes be curative^11^, but recurrence rates can vary significantly based on the severity of the disease and surgical technique^12–17^. For patients with drug-resistant epilepsy (DRE) who do not prefer, are not candidates for, or have failed resective surgery, BStim can be beneficial^18^.

Brain stimulation for epilepsy most commonly targets subcortical structures, including the thalamic nuclei, subthalamic nucleus, and caudate nucleus^19^. Reactive neurostimulation (RNS) systems have been approved by the FDA to treat epilepsy, by targeting foci with aberrant electrical activity. The 2010 “Stimulation of the Anterior Nucleus of the Thalamus for Epilepsy” (SANTÉ) multicenter trial demonstrated a 56% median reduction in seizure frequency at two years after implantation of a constant / open-loop device^20^. Long-term follow-up of patients from the SANTÉ study found a median seizure frequency reduction of 75%^21^. In 2018, the device was approved by the FDA to treat adults with epilepsy refractory to three or more antiseizure medications^18^. In 2013, the FDA approved the “RNS System” (NeuroPace RNS ® System) as the first commercially available BStim device to treat patients with specific types of drug-resistant, partial onset seizures^22^. The device, which uses computational analysis of intracranial electroencephalography (iEEG) to detect and treat active seizures, reduces seizure frequencies by 44% at 1 year and 53% at 2 years post-implantation compared to baseline measurements^22^, but with a high number of false positives (i.e., detecting a seizure that was not occurring), resulting in unnecessary brain stimulation and energy usage^23^.

Recently, researchers have leveraged ML to design closed-loop treatments for epilepsy that can modulate stimulation based on underlying neural activity. Support vector machines (SVM) have been a popular algorithm used to accomplish this task (Supplemental Table 1), as they enable efficient high-dimensional classification. In 2011, Kharbouch et al. trained an SVM to classify various types of seizures based on iEEG data (Table 2). The algorithm correctly identified seizure onset 97% of the time when tested on roughly 900 h of iEEG recordings from 10 different patients with focal epilepsy. This performance matches or exceeds the seizure detection sensitivity of trained neurologists and residents^24–26^. In addition, the algorithm’s detection delay was five seconds with a false positive rate of 0.6 per 24 h^27^. Shoeb et al. also leveraged SVM to detect seizures or epileptiform discharges based on a combination of electrocardiogram (ECG) and EEG data to direct the initiation of vagus nerve stimulation (VNS). This proof-of-concept study reported 100% sensitivity, with one false VNS every 2.5 h. Though VNS did not alter the electrographic duration of seizures, stimulation did reduce epileptiform discharges once detected^28^. The Shoeb study further validated SVM’s ability to accurately detect seizures in patients, a necessary precursor for responsive neurostimulation in a closed-loop system.

**Table 2:** Studies assessing the use of ML to develop closed-loop systems for epilepsy. Multiple ML algorithms have been leveraged and primarily used intracranial EEG as the data source.

Authors, year	Data source	Supervised, unsupervised, or computational	Key takeaways
Snyder et al.^30^	Intracranial EEG	Supervised	KNN classified data into “preictal” or “interictal” and achieved 87.5–100% prediction sensitivity.
Karbouch et al. (2011)	Intracranial EEG	Supervised	SVM detected 97% of seizures with a detection delay of 5 seconds and a false alarm rate of 0.6/24 h.
Shoeb et al.^28^	ECG and EEG	Supervised	SVM was trained to detect epilepsy and deliver vagal nerve stimulation. ECG data was used to detect heart rate changes that correspond to seizure activity.
Manzouri et al.^29^	Intracranial EEG	Supervised	SVM (AUC of 0.98) outperformed random forest (0.95) and computational line-length analysis (0.82) method used by an FDA-approved DBS device.
Zhu et al. (2020^a^)	Seizures- intracranial EEG, PD-LFPs	Supervised	The ResOT-PE model was able to detect seizures in patients with epilepsy using intracranial EEG, with a model size reduction of 3.4× and extraction cost reduction of 14.6×. The model detected a tremor in PD patients using LFPs with similar accuracy, with a model size reduction of 10.6× and extraction cost reduction of 6.8×.
Constantino et al.,^31^	Intracranial EEG	Supervised	CNN was used to predict seizures based on EEG data generated from an FDA-approved DBS device. The model achieved an AUC of 0.8–0.84, comparable to expert-level accuracy.
Liu et al., 2021	CHB-MIT scalp EEG database	Supervised	The three different machine learning algorithms were able to predict seizures from a scalp EEG database with sensitivity and FPR for the DNN, CNN, and LSTM models were 87.36%, 96.70%, and 97.61%, respectively.

^a^Study population included patients with epilepsy or PD.

Sometimes, the optimal ML algorithm used in a closed-loop BStim system depended on performance goals (e.g., low latency, high accuracy) and data bandwidth (e.g., number of EEG channels). Manzouri et al. used iEEG recordings from 10 patients with epilepsy to compare the accuracy and energy efficiency of SVM, Random Forest (RF), and the line-length algorithm used by the RNS system. In single-channel classification, RF outperformed both SVM and line length in both overall AUC (RF: 0.90; SVM: 0.88; line length: 0.83) and AUC for early detection (detection using only the first 10 seconds of iEEG data following seizure onset; RF: 0.83; SVM: 0.71; line-length: 0.73). The 17 percentage point difference between SVM’s AUC for early versus overall detection was due to longer detection latency. In multichannel classification, SVM outperformed RF and line length for overall AUC (RF: 0.95; SVM: 0.98; line length: 0.82), but still lagged behind RF for early detection (RF: 0.89; SVM: 0.84; line-length: 0.71). RF appears to outperform SVM in early seizure detection and performs comparably in overall detection. Notably, RF-based classification required a lower power microcontroller, thereby increasing energy efficiency, which is an important consideration for BStim systems^29^.

Other ML algorithms have been studied to automate the detection of epileptiform discharges. Snyder et al. proposed using the K-nearest neighbor (KNN) algorithm to classify iEEG data into “pre-“ or “interictal” and reported 87.5–100% prediction accuracy^30^. This application of KNN was to develop a warning system for upcoming seizures and would need to be paired with an electrical stimulation component to function as a closed-loop system. More recently, Constantino et al. reported using a convolutional neural network (CNN) to analyze iEEG data recorded from 22 patients implanted with the RNS system. Overall, the model achieved an AUC of ~0.8 and a mean latency of 6.3 s, which is comparable to expert epileptologists^31^. These results strongly indicate that deep learning methods such as CNN can also be effective in developing closed-loop systems to treat epilepsy.

### Movement disorders

#### Parkinson’s Disease (PD)

Classical motor symptoms of Parkinson’s disease (e.g., tremors, bradykinesia, dyskinesia, and gait disturbance) result from aberrant dopamine signaling in the thalamocortical network^32^. First-line management of PD involves dopamine replacement therapy, including monoamine oxidase inhibitors that reduce dopamine breakdown, dopamine agonists that mimic endogenous dopamine, and levodopa (L-dopa), which gets converted to dopamine systemically in the body^33^. BStim is indicated for patients with medication-refractory symptoms and/or disabling medication side effects^34^.

Similar to closed-loop applications for epilepsy, SVMs, and other non-neural network ML algorithms have shown promise for use in closed-loop systems to treat PD-associated tremors. Mohammed et al. developed SVM and Gaussian mixture models (GMM) to: (1) classify PD symptom severity in “on” and “off” L-dopa states using LFPs of the STN and (2) choose appropriate stimulation for neural modulation (Table 3). Ideal stimulation frequencies were derived from the models’ classification probabilities. Algorithm performance was compared using the Mathews correlation coefficient (MCC), which measures the correlation between observed and predicted binary classifications. MCC can be between −1 (100% disagreement) and +1 (100% agreement), with 0 representing random prediction. The MCC of prediction was >0.5 for SVM and GMM for 7 out of the 9 datasets. Overall, SVM’s MCC (median of 1) was higher than that of GMM (0.94). However, the time it took for SVM-based modulation to control LFPs was slower, with a median of 1.5 s (lower quartile: 1.25 s and upper quartile: 1.87 s), compared to the GMM system, with a median of 1.25 seconds (0.25 s and 1.75 s)^35^. These results underscore how both system characteristics (e.g., latency, settling time, power consumption, and biomarker choice) and ML model characteristics (e.g., sensitivity and specificity) must be assessed when developing closed-loop systems, as both play roles in system performance. The importance of careful biomarker selection was reported by Sand et al., who trained a feed-forward neural network and SVM using LFPs from the STNs of eight patients with PD. The models classified LFPs as being from patients in an “on” or “off” L-dopa state. The highest accuracy system was developed when using SVMs trained with individualized features selected for each patient. These individualized SVM models performed better than models trained with a common set of features, with accuracies of ~80% and ~65%, respectively^36^. Interestingly, a non-deep learning algorithm (i.e., SVM) was able to outperform a deep learning neural network, implying that higher dimensional ML model training is not always needed to achieve the best performance.

**Table 3:** Studies assessing use of ML to develop closed-loop systems for PD. Multiple ML algorithms have been leveraged and have primarily used LFPs from STN as the data source. Some solutions combine BStim systems with wearables, but most leverage BStim data alone.

Authors, year	Data source	Supervised, unsupervised, or computational	Key takeaways
Shukla et al.^40^	sEMG of forearm Accelerometer on finger	Supervised	A neural network can predict the re-appearance of tremors in a DBS “off” state and trigger the system to turn on. Overall tremor prediction accuracy was ~76%.
Niketeghad et al. (2014)	LFPs	Supervised	SVM had an accuracy of 73.2% in classifying between speech, motor, and "random" tasks. A similar algorithm could be used in a closed-loop system to identify patient behaviors to optimize DBS parameters.
Mohammed et al. (2015)	LFPs from STN in ON and OFF levodopa states	Supervised	Adaptive SVM (determines whether the linear, quadratic, or cubic kernel is better for a given data set) achieved classification accuracy >70% on 9 patients (>98% in 6 pts) in determining PD vs. non-PD state using a combination of electrodes.
Mamun et al. (2015)	LFPs from STN or GPi	Supervised	Developed a new method for feature extraction to maximize classification using LFPs. SVM and Naïve Bayes performed comparably when classifying rest vs. movement. SVM was better in determining the laterality of movement.
Golshan et al. (2016)	LFPs from STN	Supervised	Multiple kernels learning SVM outperformed single kernel SVM in classifying between button press, speech, and random movement (accuracy of 71% vs. 66%). Signals were acquired with a low sampling rate (10 Hz), thereby lowering computational cost.
Islam et al.^41^	LFPs from STN or GPi	Supervised	The ensemble model of three neural networks predicted movement with an AUC of 0.87 and laterality with an AUC of 0.86.
Mohammed et al. (2017)	LFPs from STN	Supervised	A novel dimensionality reduction technique enabled a classification accuracy of 99.3% in identifying periods of rigidity and bradykinesia.
Niketeghad et al.^38^	LFPs from STN	Supervised	The non-linear regression model asynchronously detected finger movements with an AUC of 0.7.
Shah et al. (2018)	LFPs from STN	Supervised	The LR-based classifier achieved AUC ranging from 0.67 to 0.93 in predicting resting tremors in PD patients.
Golshan et al. (2018)	LFPs from STN	Supervised	The SVM classifier achieved an accuracy of 85% in predicting behaviors such as "reach" and "press button" in patients on and off PD medications.
Camara et al. (2019)	LFPs from STN	Supervised	Recurrence networks identified the onset of non-linearities in the STN to predict the transition between movements and anticipate the onset of tremor.
Yao et al. (2020)	LFPs from STN	Supervised	Kalman filtering of LFPs from STN improves the specificity of ML algorithms in detecting PD resting tremors by 17%.
Castaño-Candamil et al.^44^	Neural markers from EEG readings	Supervised	Patient-specific band power neural markers from EEG data were able to better decode hand movement in PD patients with DBS than standardized markers alone.
Kuhner et al. (2020)	3D gait data from inertial measurement units	Supervised	AdaBoost could classify movements as abnormal across patients with PD and normal subjects. Data from the foot and lower leg segments were most useful in classifying abnormal movements, and variability of smoothness (e.g., jerk of movement) was the most useful feature in classification.
Golshan et al. (2020)	LFPs from STN	Supervised	A deep CNN-based model was 88% accurate in classifying actions (e.g., reach, press, and speech) in PD patients and used fewer input parameters compared to other models in the literature.
LeMoyne et al. (2020)	Inertia sensor (*BioStamp nPoint*) on dorsum of hand	Supervised	A neural network used data from the *BioStamp nPoint* inertia monitoring device and achieved 95% accuracy in distinguishing between various DBS amplitude settings.
Mohammed et al.^35^	LFPs from STN	Supervised	SVM performed better than a Gaussian Mixture Model in classifying patients in PD versus non-PD and quantifying symptom severity in the PD group. Both models suppressed PD symptoms in 7/9 cases in under 2 s.
Ahn et al.^37^	Neural activity from STN	Supervised	Support vector regression was able to track a PD symptom severity metric even at a one-second timescale.
Khawaldeh et al.^39^	LFPs from STN	Supervised	Naïve Bayes enabled prediction of intended movements after patients knew the movement, but before they were cued to move.
Sand et al.^36^	LFPs from STN	Supervised	Highest accuracy (80%) in classifying patients into an “on” or “off” levodopa state comes from an SVM trained on features specific to each patient. Models trained using standard beta and gamma wave activity had accuracies of 64–66%.

Another group of researchers leveraged SVM for regression rather than classification. Ahn et al. used support vector regression (SVR) to track a novel measure of PD symptom severity called the “Motor Error Score” (MES), which measures the difference between a defined motor activity and a PD patient’s execution of that activity. SVR was trained using neural activity from the STN and was able to predict MES with an accuracy range of 0.14–0.88, depending on how much data was given to the model. Applying SVR and similar techniques in this manner could help link neural activity to PD symptom severity, thereby providing an avenue for closed-loop BStim^37^. Similar regression techniques have also been used for movement detection. Using LFPs of patients with PD who were executing a button-pressing task, Niketeghad et al. developed a non-linear regression model that could asynchronously detect finger movements with an AUC of 0.7^38^. Methods similar to this could be used to predict/detect tremors or other irregular movements, thereby triggering neural modulation.

Non-regression methods have also shown promise for application in movement detection for closed-loop systems. Khawaldeh et al. trained a naïve Bayes (NB) classifier with LFP data from the STN of patients with PD as they executed upper and lower limb movements^39^. When patients knew the action ahead of time, the NB algorithm had an AUC of 0.8 in predicting movements before a cue to move was given. Specifically, activity in the alpha and beta frequency bands contributed significantly to the algorithm’s prediction accuracy. Similar algorithms could be used to predict abnormal movements and deliver prophylactic neural modulation.

The performance of the artificial neural network-based closed-loop systems to treat PD somewhat lags that of non-neural network-based systems. Shukla et al. developed a feedforward neural network that used sEMG of the forearm and accelerometer data from fingertips to predict the re-emergence of tremors following the removal of stimulation, but before patients experienced discomfort. Spectral characteristics of the sEMG and accelerometer data were used as inputs to the model, resulting in an accuracy of ~76% and sensitivity of ~92% in predicting tremor re-emergence^40^. Other researchers have developed artificial neural networks that use LFPs to make predictions. Islam et al. developed an ensemble model comprised of (1) standard feedforward neural network, (2) a radial basis function neural network, and (3) a probabilistic neural network to first differentiate movement from resting state and then decode laterality in patients with PD or dystonia. Spectral features of LFP data from the STN or globus pallidus internus were used as inputs to the networks. The model’s final classification was determined based on majority voting among the 3 networks, resulting in an AUC of 0.87 for movement and 0.86 for laterality classification. Ensemble model performance was superior to that of any individual neural network^41^. Refining such models that differentiate PD tremors from general movements will increase their utility in closed-loop BStim. Efforts in this regard have used CNNs to directly analyze graphical LFP data rather than extracting features from the data. Yao et al. trained a CNN to detect a resting tremor in patients with PD and reported an F1 score of ~77%. Though CNN was not the highest-performing model tested, its performance shows promise for future application in closed-loop systems^42^.

#### Essential tremor

Essential tremor (ET) is one of the most common causes of action tremor, which appears with voluntary muscle movement. While there are currently no disease-modifying treatments available for ET, symptom management can be achieved through the use of beta-blockers and anticonvulsants^43^. Patients also self-medicate with alcohol. Closed-loop therapies are particularly relevant for ET, since tremor almost exclusively occurs in specific motor contexts.

Standard regression techniques have been successfully implemented for the closed-loop treatment of ET. Castaño-Candamil et al. developed a linear regression model that used iEEG data from the primary motor cortex to estimate tremors and generate neural stimulation (Table 4). Correlations between estimated and true tremor intensities varied from 0.21 to 0.39. The system also reduced power consumption by 24–80% and performed better than open-loop in 2 out of 3 sessions as assessed by the Fahn-Tolosa-Marin rating scale used to assess tremor intensity in patients with ET^44^.

**Table 4:** Studies assessing the use of ML to develop closed-loop systems for ET. Logistic regression has commonly been used when analyzing ET. Many closed-loop systems achieved tremor suppression equivalent to traditional BStim.

Authors, year	Data source	Supervised, unsupervised, or computational	Key takeaways
Shukla et al., 2014	sEMG of forearm Accelerometer on hand	Supervised	A decision tree-based algorithm predicted the reappearance of tremor during a DBS “off” state with an accuracy of 93%. The algorithm then switched DBS to the “on” state.
LeMoyne et al.^49^	iPhone on hand	Supervised	SVM achieved 100% accuracy in classifying DBS “on” versus “off” states.
Khobragade et al., 2018^a^	sEMG and accelerometry signals from the arm	Supervised	The proposed algorithm achieved a maximum of 100% sensitivity in predicting the onset of tremors after cessation of DBS stimulation
Houston et al.^45^	Intracranial EEG	Supervised	Tremor suppression of logistic regression (LR)-based closed-loop DBS system was comparable to continuous DBS.
Tan et al.^46^	LFPs from ViM thalamus	Supervised	LR-based closed-loop DBS system was able to predict the occurrence of postural tremors with an AUC of 0.88.
Tan et al.^46^	LFP from ViM thalamus, EMG	Supervised	The LR model identified postural tremors and voluntary movements during DBS surgery and after surgery, respectively. The average sensitivity was 0.8, with a false detection rate of 0.2.
Castaño-Candamil et al., 2021	Intracranial EEG IMU on the wrist	Supervised	Linear regression predicted tremor intensity from EEG data with correlations from 0.21 to 0.39. The system performed better than continuous DBS in reducing tremors in two of three cases.
Opri et al.^47^	Subdural cortical electrodes	Supervised	Closed-loop system based on an LDA classifier identifying tremor-producing states achieved tremor suppression on par with continuous DBS systems.
He et al.^48^	LFPs from ViM thalamus	Supervised	SVM had an accuracy of ~80% in detecting tremor-provoking movement states.
Fuchs et al., 2021	A smartphone on the wrist to measure acceleration and rotation	Supervised	The model was able to successfully assess tremor severity in patients with ET with a mean absolute error of 78% lower than linear models and 71% lower than decision tree models.

^a^Study population included patients with ET or PD.

Many groups have used logistic instead of linear regression for ET classification. Houston et al. leveraged logistic regression to identify “tremor-provoking movements” based on iEEG data from the hand/arm area of the primary motor and somatosensory cortices. Outputs of the model were then used to influence stimulation intensity. The model was evaluated during both a prompted movement (PM) task comprised of movements that evoke tremor and a natural movement task (e.g., drawing and writing). The model was 75% accurate in the PM task and 85% accurate in the natural movement task. Overall, this closed-loop system did not significantly improve tremor compared to continuous stimulation, though further validation must be conducted with increased sample sizes and data bandwidth^45^. Tan et al. (2019) also used logistic regression trained on LFP data from the ventral intermediate (ViM) thalamus to predict postural tremors. This model had an AUC of 0.88 and detection latency between −0.4 and +0.3 s, indicating the classifier could predict tremor onset^46^.

Linear discriminant analysis (LDA) has been another popular algorithm in closed-loop BStim for ET. Opri et al. used LDA on spectral features from low-frequency oscillations recorded from the primary motor cortex during voluntary movements. The lowest-performing LDA classifier had an accuracy of ~86%, with accuracies reaching 93% when the model was trained using three months of data. Overall, the LDA-based closed-loop system achieved tremor suppression comparable to continuous stimulation while reducing the amount of stimulation delivered^47^. In 2021, He et al. detected “tremor-provoking states” using ML models trained using time and frequency domain characteristics of LFPs from the ViM thalamus. SVM performed the best, with an accuracy of ~84% during real-time testing, though LDA was among the top-performing algorithms. This system reduced tremor to the level of continuous stimulation systems but delivered ~60% less energy via stimulation^48^.

SVM has performed well in other attempts to create non-neural signal-based closed-loop systems for treating ET. LeMoyne et al. developed an SVM classifier using data from a smartphone-based accelerometer to identify patients receiving brain stimulation. The SVM achieved 100% accuracy using spectral tremor features from the smartphone placed on the dorsum of patients’ hands^49^. Similar classifiers that leverage tremor information from smart devices (e.g., phones and watches) are an alternative to neural signal-based classifiers in creating closed-loop systems.

Non-SVM algorithms have also utilized tremor information from wearable devices. Shukla et al. used decision trees trained on sEMG signals from the forearm and accelerometer data from the hand. The model used 2 consecutive decision tree classifiers. First, the model classified whether patients were in the movement or postural (e.g., hands outstretched) condition. Depending on the first classification, the model used two separate decision trees to predict whether tremor would re-appear in a stimulation “off” state and initiated stimulation if needed. The model achieved an accuracy of ~93% with a ~3% false alarm rate. The predicted time of tremor re-appearance was only ~7% different from the actual time of re-appearance, thereby enabling the model to promptly initiate stimulation when needed^50^.

#### Tourette’s syndrome

Tourette’s syndrome (TS) is a neurological disorder characterized by sudden and repetitive motor and vocal tics that likely arise due to abnormal neural signaling in the mesolimbic pathway. Management of TS is multifaceted, including both behavioral therapy and pharmacologic intervention with alpha-adrenergic agonists and anti-dopaminergic medication^51^. BStim is still being investigated as a treatment for TS, though the benefit of closed-loop systems is clear, as they would enable pre-emptive neural modulation to prevent the appearance of tics.

Initial work studying BStim to treat TS has focused on establishing accurate biomarkers that can be used to track the progression/severity of the disease. Marceglia et al. used LFPs from the thalamus of patients with TS to characterize neural activity corresponding to tics (Table 5). They report that tics are preceded by a ~20% decrease in alpha (8–13 Hz) band activity, followed by a ~150% increase in both LF (2–7 Hz) and alpha band activity. Interestingly, voluntary movements follow different patterns of neural activity. These findings could be used to differentiate between tics and voluntary movements when developing closed-loop systems.

**Table 5:** Studies assessing the use of ML to develop closed-loop systems for Tourette’s.

Authors, year	Data source	Supervised, unsupervised, or computational	Key takeaways
Marceglia et al., 2018	LFPs from thalamus	Computational	LFPs corresponding to tics follow specific patterns (e.g., preceded by ~20% decrease in alpha band activity) that can be used to differentiate them from voluntary movement.
Neumann et al., 2018	Pallidal and thalamic beta and theta	Supervised	Multivariate linear regression could predict Yale Global Tic Severity Scale (YGTSS) scores with an *r*^2^ of 0.96.

Other groups have applied regression techniques on similar LFP data to predict TS severity. Neumann et al. applied multivariate linear regression using LFPs from pallidal and thalamic electrodes to predict Yale Global Tic Severity Scale scores in patients with medication-refractory TS. The regression had an *r*^2^ coefficient of 0.96, suggesting that LFP data can inform disease severity in TS. More research is required to understand how LFP data can be decoded to provide real-time estimates of TS manifestation and severity.

### Neuropsychiatric diseases

There is increasing evidence that neuropsychiatric diseases are network disorders of neuronal signaling^52,53^. Evidence for the network basis of depression is well known, especially with respect to the role of GABAergic and serotonergic systems^54,55^, and there is longstanding evidence regarding the role of various neurotransmission networks throughout the brain in the development and progression of schizophrenia^56–58^. Similar data have emerged for the network basis of obsessive–compulsive disorder^59–62^. As such, these neuropsychiatric disorders could be investigated for potential treatment with BStim. Instead of monitoring changes in motor symptoms, treatment effectiveness needs to be assessed with reductions in mood or psychiatric manifestations.

#### Depression

Depression is a complex condition with a diverse etiology, though the neural network model of depression has gained support through postmortem studies. Specifically, data supports aberrant serotonergic and GABAergic circuits in multiple locations, including the anterior cingulate, amygdala, nucleus accumbens, and prefrontal cortex^63^. Initial treatment for depression is variable and can include behavioral therapy and/or medications such as selective serotonin reuptake inhibitors^64^. Given the strong evidence underlying the network disease hypothesis of depression, BStim is being investigated as a treatment option and has shown varying degrees of success in targeting different parts of the brain^65,66^.

Research into neural biomarkers that could be used to develop closed-loop systems for depression is relatively new. However, research efforts have focused on developing and testing different machine learning models based on varied training data sets (i.e., EEG, vitals signs from smartwatches). In 2021, Hopman et al. evaluated multiple ML algorithms, including decision trees, SVM, logistic regression, and NB, on their ability to predict long-term responses to repetitive transcranial magnetic stimulation (rTMS) in patients with medication-refractory depression (Table 6). Biomarkers related to functional connectivity measurements derived from fMRI data were used to train the models. Linear SVM displayed the best overall performance in the study, achieving AUC greater than 0.9 and accuracies as high as 95%^67^. These models, though not specifically trained on data related to BStim, highlight the role of ML in leveraging neural biomarkers.

**Table 6:** Studies assessing the utility of various biomarkers for monitoring neuropsychiatric disorder symptom severity. Most work has focused on using EEG data, though some work has started analyzing LFPs as well.

Indication	Authors, year	Data source	Supervised, unsupervised, or computational	Key takeaways
Depression	Neumann et al.^87^	LFPs from the stria terminalis and cingulate cortex	Computational	Alpha activity (8–14 Hz) in the bed nucleus of stria terminalis correlated with the severity of depressive symptoms.
	Wickramasuriya et al., 2019	Skin conduction	Computational	Skin conduction can be used to assess sympathetic activity, which has been shown to be abnormal in depression
	Hopman et al.^67^	Functional connectivity from fMRI	Supervised	SVM was used to predict long-term responses to repetitive transcranial magnetic stimulation (rTMS) and achieved accuracies as high as 95%.
	Shah et al.^68^	Smartwatch EEG Mobile app	Supervised	The ensemble model generated predictions of depressed mood and had an error of 29.7%. The best model (e.g., SVM, random forest) differed for each patient.
	Uyulan et al.^69^	EEG	Supervised	A neural network achieved accuracies between ~84 and ~96%, while a CNN achieved an accuracy of ~90% in diagnosing depression.
	Movahed et al.^70^	EEG	Supervised	SVM classified patients with depression with an average accuracy of 99% and a false discovery rate of 0.4%.
	Sendi et al.^71^	LFPs from subcallosal cingulate (SCC)	Supervised	Logistic regression had an AUC of 0.73 in distinguishing between pre-and post-DBS LFPs from the SCC in patients with treatment-resistant depression. The SCC LFP beta band was most correlated with improvements in clinical symptoms.
Schizophrenia	Kim et al.^82^	EEG	Supervised	An LDA classifier had an accuracy of ~88% when classifying between high and low-severity SCZ with predominantly positive symptoms.
	Trajkovic et al.^81^	EEG	Supervised	SVM and logistic regression classifier differentiated between high and low schizotypal groups with a combined AUC of 0.83.
	Masychev et al.^80^	EEG	Supervised	SVM, random forest, and Gaussian NB all achieved an accuracy of ~92.7% in differentiating SCZ from healthy controls.
	Zhao et al.^79^	EEG	Supervised	SVM achieved an accuracy of 95.2% when classifying patients with SCZ and healthy controls.
Obsessive–compulsive disorder	Aydin et al.^90^	EEG	Supervised	SVM differentiated between patients with OCD and healthy controls with an accuracy of 85%.
	Takagi et al.^88^	Connectivity from fMRI	Supervised	A logistic regression-based algorithm classified OCD versus healthy controls with an AUC of 0.7.
	Rappel et al.^85^	LFPs from STN	Computational	OCD symptoms are inversely correlated with theta activity (6.5–8 Hz) in the ventral STN.
	Ding et al.,^89^	Facial affect recognition	Supervised	Both gradient boosted decision trees and SVM differentiated between pre- and post-DBS adjustment states with a maximum F1 score of 0.76.
	Smith et al., 2020	LFPs and EEG	Computational	The connectivity between midfrontal lobe EEG and ventral capsule/ventral striatum LFPs correlated with baseline and posttreatment OCD symptoms.
	Metin et al., 2020	Power features from quantitative EEG	Supervised	Neural networks that used theta band (4–7 Hz) features predicted patient response to rTMS with 80% accuracy.

Shah et al. compared the efficacy of random forest, gradient boosting, Ada boost, SVM, and other algorithms in generating predictions of depressed mood over a 1 month time period. The models were trained on a variety of data, including in-lab EEG recordings, heart rate information from smartwatches, mood ratings, stress assessments, and diet reporting from a mobile app. When evaluated on mean absolute percentage error, an ensemble model performed the best in a predicting mood, with an error of 29.7%. However, the best model differed for each patient, highlighting the highly individualized nature of these predictions. In that regard, feature importance analysis revealed variation from patient to patient, but certain features (e.g., anxiety level, physical activity levels, and diet logs) were often in the top 5 most important features across all patients^68^.

Other groups are leveraging more traditional biomarkers to assess their potential for informing depression severity. Uyulan et al. developed a feedforward neural network and CNN to diagnose depression based on EEG data. Features derived from frequency domain analysis of EEG signals were used to train the feedforward network, achieving accuracies between ~84 and ~96%. The CNN was a combination of the popular ResNet50 convolutional network and a long-short-term memory (LSTM) network added to capture information in the time-dependent EEG. This CNN achieved an accuracy of ~90%^69^. Movahed et al. also developed models to analyze EEG data but focused on non-neural network-based algorithms. The group tested SVM, logistic regression, decision trees, NB, and other ensemble classifiers trained using features extracted from spectral analysis of EEG data. Overall, SVM with a radial basis kernel performed the best in classifying patients with depression, with an average accuracy of 99% and a false discovery rate of 0.4%. NB had the lowest classification accuracy of 87%^70^. Recent work by Sendi et al. has further advanced biomarker development for depression. The group assessed intraoperative LFPs from the subcallosal cingulate (SCC) in patients with treatment-resistant depression undergoing bilateral BStim and found that declines in depressive symptoms after treatment correlated with reductions in SCC LFP beta power (13–30 Hz)^71^. Further exploration of stimulation targets across cortical and subcortical structures has shown promising results in preliminary studies^72^.

#### Schizophrenia

Schizophrenia (SCZ) is a complex psychiatric illness that presents with a combination of positive (e.g., hallucinations) and negative (e.g., flat affect) symptoms that lead to severe functional impairment. SCZ is hypothesized to arise due to improper signaling of several neurotransmitters, including dopamine, glutamate, and acetylcholine^73,74^. Patients with SCZ have traditionally been managed with psychosocial interventions and antipsychotic medications. In the past few years, more research has been conducted to identify specific areas within the brain that could be targeted for BStim in treating SCZ^75–77^. Recently, a small-scale clinical trial showed promise for BStim in improving SCZ severity^78^. However, significant work is still needed to identify neural biomarkers and thus improve the efficacy of BStim for SCZ.

Non-neural network ML algorithms have played a large role in the classification of SCZ. In 2021, Zhao et al. used an SVM trained on EEG spectral features and measures of connectivity to classify patients with SCZ and healthy controls (Table 6). The model performed well, achieving an accuracy of 95.2%, specificity of 94.4%, and sensitivity of 96.2%. Of note, the SVM achieved this level of performance after using a feature set that combined two different, but complementary, methods of analyzing neural connectivity^79^. Masychev et al. also looked to differentiate SCZ from healthy controls based on EEG data and connectivity measurements. Interestingly, three algorithms are tied for the best performance. SVM, random forest, and Gaussian NB all achieved an accuracy of ~92.7%. An LDA classifier achieved an accuracy of ~90.2%^80^.

SVM has also been applied to differentiate between disease severity in SCZ. Trajkovic et al. used spectral features of resting state EEG along with measures of intrahemispheric connectivity to train both an SVM and logistic regression classifier to differentiate between high and low schizotypal groups as defined by the Schizotypal Personality Questionnaire. The models achieved a combined AUC of 0.83, implying that EEG data could be used to assess disease severity in patients with SCZ^81^. Kim et al. used similar features to train an LDA classifier combined with feature selection. The highest classification accuracy of ~88% occurred when classifying between high and low-severity SCZ with predominantly positive symptoms. Classifying between high and low severity with predominantly: (1) negative symptoms had an accuracy of ~75%, and (2) cognitive/disorientation symptoms had an accuracy of ~78%^82^.

#### Obsessive–compulsive disorder

Obsessive–compulsive disorder (OCD) is characterized by intrusive thoughts (obsessions) that drive repetitive actions/rituals (compulsions). Though the specific etiology of OCD is still being investigated, current data implicate multiple neuronal circuits, including the cortico-striato-thalamo-cortico and fronto-limbic circuits^83^. Treatment for OCD typically involves therapy and medications (e.g., selective serotonin reuptake inhibitors)^84^. Similar to depression, research on using BStim for OCD has focused on identifying potential targets in the brain, with most literature on the striatal region and dorsal STN^85–87^. Still, ideal anatomical targets and disease biomarkers have yet to be found.

Some researchers have used non-LFP biomarkers to classify patients with OCD. In 2017, Takagi et al. used connectivity features extracted from resting state fMRI to train a logistic regression-based algorithm to classify OCD versus healthy controls (Table 6). When tested on an external data set, the algorithm achieved an AUC of 0.7^88^. Another group applied gradient-boosted decision trees and SVM on facial recordings of patients with OCD to differentiate between pre- and post-stimulation adjustment states. By analyzing various facial landmarks, Ding et al. found that both gradient boosted decision trees and SVM differentiated between pre- and post-stimulation adjustment states with a maximum F1 score of 0.76. However, gradient-boosted decision trees utilized fewer features to achieve the same F1 score^89^.

Many research groups have applied ML algorithms for EEG analysis in the context of OCD. Aydin et al. trained an SVM using single-channel EEG data from patients with OCD and reached a classification accuracy of 85% when differentiating between OCD and healthy controls^90^. In 2020, Metin et al. used power features from 19 nodes in quantitative EEG as inputs to a feedforward neural network that predicted patient response to rTMS. Using theta band (4–7 Hz) power features as inputs to the network led to 80% accuracy. Overall, these studies show the potential of machine learning algorithms to analyze neural data and make predictions about disease characteristics in patients with OCD.

## Machine learning integrations and future directions

**Fig. 1:**
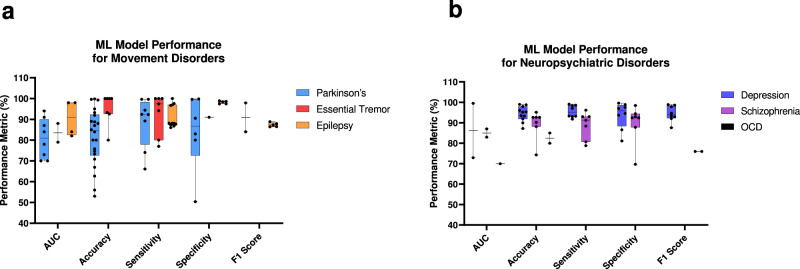
Aggregated ML model performance. **a** ML model performance metrics were reported across 39 unique papers assessing ML in closed-loop systems for movement disorders. The models generally exceed 80% across performance metrics. Significant variation in model performance exists even for historically well-studied diseases such as Parkinson’s. More data is needed for essential tremors. **b** ML model performance metrics reported across 17 unique papers assessing ML in closed-loop systems for neuropsychiatric disorders. The median performance for depression and schizophrenia generally exceeds 90%. There is a lack of data on OCD. All data is shown as median (center line) and first and third quartiles, with bars representing minimum and maximum.

The literature regarding ML in closed-loop systems is relatively new, but the research assessed here represents a strong beginning to realizing the potential of ML applications in the field. Certain diseases (e.g., Parkinson’s) have been studied more compared to others (e.g., SCZ), and, in general, movement disorders have more data regarding ML model performance compared to neuropsychiatric disorders. Regardless, aggregated ML model performance across studies reveals significant inter-study variation (Fig. 1). Though the median model performance was generally greater than 80% across most metrics, some studies report much lower performance. Interestingly, large performance variation is most notably seen for Parkinson’s, which is the best-studied disease with respect to ML and closed-loop systems. Depression had the highest median performance across metrics, and more data is needed for essential tremor and OCD (Fig. 1). Notably, most publications assessed here that studied neuropsychiatric disorders primarily focused on biomarker evaluation and did not study fully developed closed-loop systems. Continued research with larger sample sizes will play a role in reducing variability and improving model performance. Of the studies included here, ~82% had fewer than 30 participants (Supplemental Fig. 1). Combining data sets across institutions, improving post-publication data-sharing protocols, and creating data repositories could help address this issue.

As more work is done to optimize the design of ML models in closed-loop systems, ML algorithm choice will become increasingly important. With significant implications for model accuracy, speed, and energy usage, choosing a specific ML algorithm (e.g., neural network vs. random forest vs SVM) for a task and dataset can be challenging and still generally requires trial and error. There is significant research being conducted to determine how to best predict optimal algorithms and model parameters (e.g., learning rates for gradient descent)^91–94^. In reality, many different ML algorithms could be effective in performing a task on a specific dataset^95,96^. High throughput applications of ML have favored using neural networks as they are able to derive complex, non-linear relationships in a relatively efficient manner, though they can be “black box” and lack in ease of interpretability^97–99^. Convolutional neural networks have also been leveraged in closed-loop applications, as they enable deep learning without requiring feature engineering since image data itself (e.g., graphs of LFPs) are used as model inputs. In addition, the use of image data enables synthetic data enhancement to augment small datasets. Commonly used non-neural network algorithms in the papers analyzed here include SVM and decision trees. While decision trees tend to overfit training data, groups have leveraged “boosting” or “bagging” techniques to reduce bias and variance. It is unlikely that one ML algorithm will emerge as the “best” for closed-loop BStim applications, and, as seen here, different model designs will have varying success for different use cases.

## Discussion

Machine learning is emerging as a crucial tool in the design of closed-loop BStim systems. Researchers are creating closed-loop systems that integrate neural and non-neural network-based machine learning algorithms to address both established (e.g., PD) and newer (e.g., OCD) indications. In diseases, such as PD, for which neural pathophysiology is better understood, most research has focused on refining algorithms to improve the classification of neural signals as aberrant or normal and then initiate proper stimulation, though work related to biomarker identification has continued. In other conditions, such as many neuropsychiatric disorders, the underlying abnormal neural circuitry is still being understood, and research efforts are primarily focused on discovering biomarkers (e.g., LFPs from brain nuclei) to identify the onset of disease-specific symptoms and stratify the severity of disease. Regardless of the specific application, closed-loop systems have promise in managing symptom severity to an extent that medications and traditional brain stimulation are unable to achieve.

The NeuroPace responsive neurostimulation system (RNS) was the first FDA-approved closed-loop technology and, as such, serves as an important case study for future development in the field. Approved for cases of uncontrolled seizures localized to one or two epileptogenic foci, the RNS consists of a cranially-seated neurostimulator with leads placed at seizure foci. There have been multiple clinical trials showcasing the efficacy of the RNS in reducing seizure intensity and frequency^23,100–102^. At the 9-year follow-up of 230 patients with the RNS, 73% of patients were responders with a median reduction in seizure frequency of 75%^102^. The RNS serves as a model of comparison as the first FDA-approved device in this field and helped establish the utility of closed-loop systems, but also helped identify opportunities for improvement. Specifically, future technologies building off the RNS could reduce false positives, improve detection latency, and increase the personalization of detection and stimulation.

BStim is following the overall trend in healthcare towards personalized medicine. The future of neural modulation will likely involve closed-loop systems that can predict the onset of symptoms (e.g., tremors and hallucinations) and provide stimulation tailored to ensure that patients never experience symptoms. These closed-loop systems will also further enable understanding of diseases in context, by helping gather data about disease manifestations outside of healthcare settings and in naturalistic states. This increased breadth of data collection will also allow for self-enabled ML model improvement within and between patients, as data from novel environments the model has not encountered during training can be used to improve performance. Preliminary work in this regard has been conducted on focal epilepsy^103^, but more is needed across other diseases. An added layer of complexity arises when neural stimulation is used to attenuate both motor and non-motor symptoms. As the majority of current BStim applications are for motor symptoms, the role of these systems in improving non-motor or cognitive symptoms is yet to be elucidated^104^. Some have found that targets traditionally modulated to control motor symptoms may also influence non-motor functions such as expressing vocal emotion^105^ and processing facial expression^106^. As novel and improved targets for modulating psychiatric symptoms emerge, BStim may need to increase coverage within the brain, change modulation based on symptom type, and become more individualized. Complex neuropsychiatric disorders that are more associated with non-motor symptoms have multiple foci of aberrant activity within the brain, which might all need to be modulated to control symptom severity^107^. This increasing coverage also compounds the potentially harmful effects of false positives that lead to unnecessary neural stimulation. Modulating non-motor symptoms of neuropsychiatric disorders will also require understanding variations in neural signals causing different types of symptoms (i.e., hallucinations, cognitive impairment, and flat affect) and altering stimulation accordingly. Finally, with person-to-person variation in the structure and importance of targets in neuropsychiatric disorders^108,109^, closed-loop stimulation will require increased personalization in the form of more up-front mapping of neural circuitry and post-implantation fine tuning^110^. The complexity of target choice has recently been highlighted through RNS-mediated treatment of idiopathic generalized epilepsy, which generally does not have specific epileptiform foci in the brain, yet has responded to modulation of the centromedian nucleus^111,112^.

Realizing a future in which closed-loop systems can be used to treat multiple motor and non-motor indications faces three main barriers. First is the identification of reliable biomarkers that can be used to predict/assess when a patient is experiencing symptoms. The majority of biomarkers identified to date have been related to neural activity. For example, LFPs from thalamic nuclei have been suggested to determine when a patient with PD is experiencing tremors. These biomarkers are complementary to BStim systems, as the electrodes implanted for stimulation can also be used to read neural activity. In an ideal scenario, the biomarkers used to train closed-loop systems are consistent across patients and can be used to predict the onset of symptoms or have short enough latency such that patients only experience symptoms for a brief period of time before BStim has attenuated them. Before closed-loop BStim can be a viable option for conditions like depression, SCZ, and OCD, more research must be conducted to identify disease-specific and sensitive biomarkers^75^. Even in established use cases for BStim, continued investigation into improving the safety of targets is needed to prevent adverse events such as stimulation-induced cognitive impairment^113^. Recently, there has been increasing attention paid to using data from fMRI/neural connectivity studies in designing closed-loop systems. Boutet et al. studied fMRI’s role in assessing responses to BStim in patients with PD and reported that fMRI could be used in the future to fine-tune stimulation settings^114^. Similar findings have been reported across focal epilepsy^115^ and OCD^116,117^. The future role of fMRI/neural connectivity in closed-loop systems will likely be focused on operative planning and stimulation refinement. Finally, more work is needed to understand the potential role of biomarkers derived from wearable devices in BStim for movement disorders. For example, accelerometer data from smartwatches could be used in conjunction with neural signals from deep brain structures to better inform modulation.

Closed-loop systems that use neural biomarkers to make stimulation decisions also face the issue of stimulation response latency. Since standard electrodes cannot sense LFPs and provide electrical stimulation at the same time, the use of these electrodes requires sequential steps of aberrant biomarker detection → stimulation → response detection. This can be problematic as the symptoms of many neurological disorders have short latency between neural and physical manifestations. Therefore, using traditional electrodes in closed-loop systems renders the closed-loop system blind to variations in neural activity during stimulation^118^, risking the potential for over- or under-treatment. Bi-directional electrodes have emerged as a solution for this problem, as they enable concurrent, real-time sensing and stimulation. Early research in the development of such electrodes involved determining optimal designs for filtering stimulation-mediated electrical noise^119^. While this work continues, bi-directional electrodes are becoming commercially available (e.g., *SenSight* from Medtronic), and initial testing in human^120–122^ and ovine^123,124^ models has been promising. The ML models already developed for closed-loop systems discussed here can eventually be applied to these bi-directional electrodes, and the software-hardware combination will further enable more dynamic neural modulation. Future work in this space can focus on improving the speed of signal-stimulation synchronization and reducing the impact of stimulation-induced noise on signal sensing.

The third main barrier to developing clinically viable closed-loop BStim systems is the performance of machine learning algorithms. Machine learning-based closed-loop systems must achieve levels of performance equivalent to or exceeding open-loop BStim while maintaining similar or better safety profiles. Achieving this performance goal will require developing algorithms that can identify and stratify disease with a similar proficiency to trained clinicians. While some algorithms have reached or are approaching this level of performance, there needs to be more consistency. Getting to this point will require further translational and clinical research that can aggregate and analyze large volumes of patient data. Fortunately, multiple efforts are being made in this regard^125–128^. Apart from ML-based detection of abnormal neurological activity, closed-loop systems of the future could also leverage ML for personalized treatment modulation^129^. In this scenario, ML models would assess post-stimulation biomarker activity (e.g., LFPs, tremor recorded from smartwatch) and learn optimal stimulation characteristics (e.g., frequency, duration) for specific patients. Continued work to address barriers involving ML model design is vital to realizing the full therapeutic potential of what closed-loop BStim could deliver.

## Methods

A literature review was performed in three steps. First, a manual search was conducted using PubMed with the search terms: “*machine learning AND deep brain stimulation”* and “*machine learning AND (adaptive deep brain stimulation OR closed-loop deep brain stimulation)”*. Another review was conducted in PubMed and Embase using unique Medical Subject Headings (MeSH) terms associated with the articles from the manual search. Finally, a third search was conducted in PubMed with the search terms “closed-loop deep brain stimulation AND *disease name*” (e.g., *“closed-loop deep brain stimulation AND schizophrenia”*). The abstracts of all articles were reviewed to determine inclusion. Inclusion criteria included the use of machine learning (i.e., an algorithm designed to make predictions on new data given a set of training data), human participants, and the study of a biomarker that could be used for closed-loop feedback. Studies that were not original research articles (e.g., reviews, editorials) or those that did not specifically evaluate closed-loop systems were excluded. A total of 783 articles were evaluated, yielding 56 unique articles after applying inclusion and exclusion criteria (Supplemental Fig. 2).

## Supplementary information

Supplemental Information

## Data Availability

The data used to generate the tables are publicly available to researchers through the National Library of Medicine. Additional inquiries are welcome to the corresponding author.
