# Supplementary material for: Landscape and future directions of machine learning applications in closed-loop brain stimulation
Source: NPJ Digit Med. 2023 Apr 27;6:79. doi: 10.1038/s41746-023-00779-x (PMC10140375; doi:10.1038/s41746-023-00779-x)
Supplement: Supplementary file 1 — Supplemental Information [file 41746_2023_779_MOESM1_ESM.pdf]

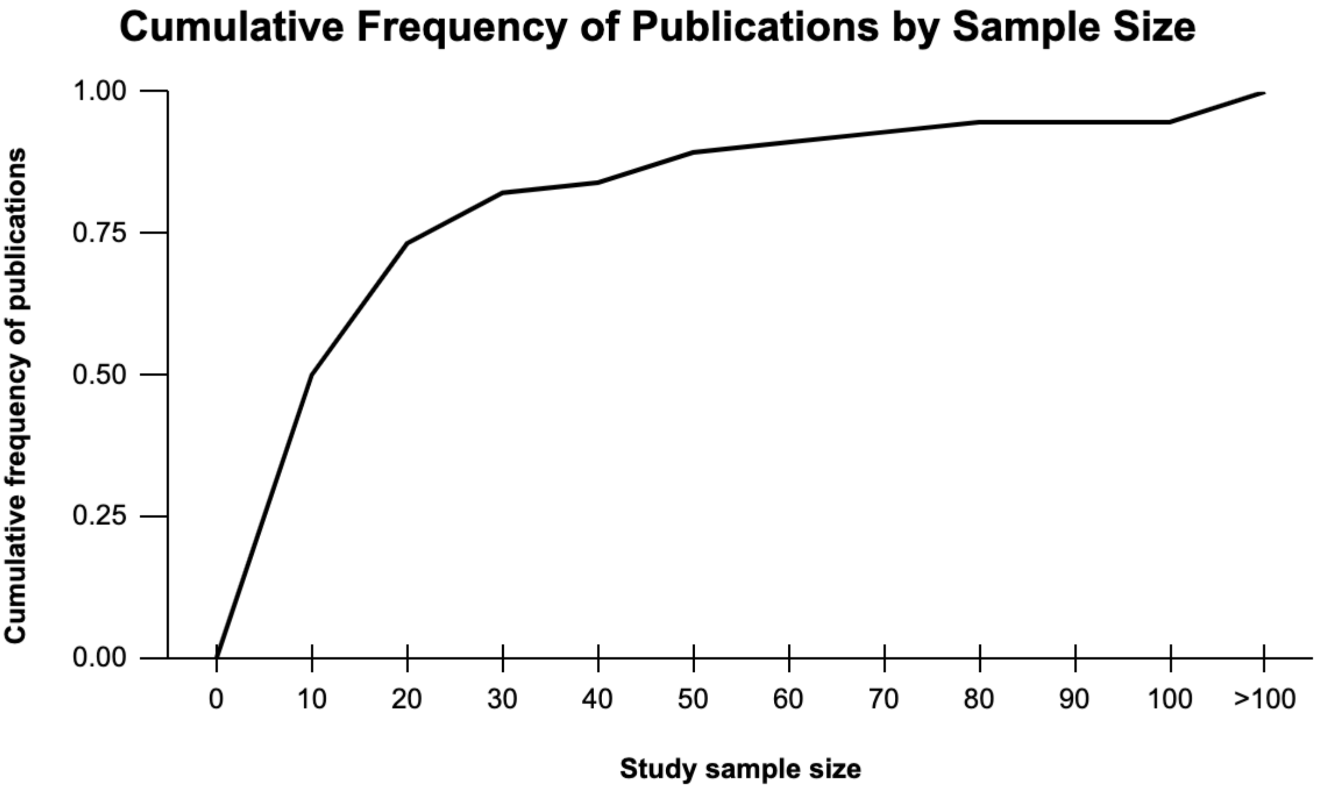

Supplemental Figure 1. Cumulative frequency of publications by study size. 82% of studies had fewer than 30 participants.

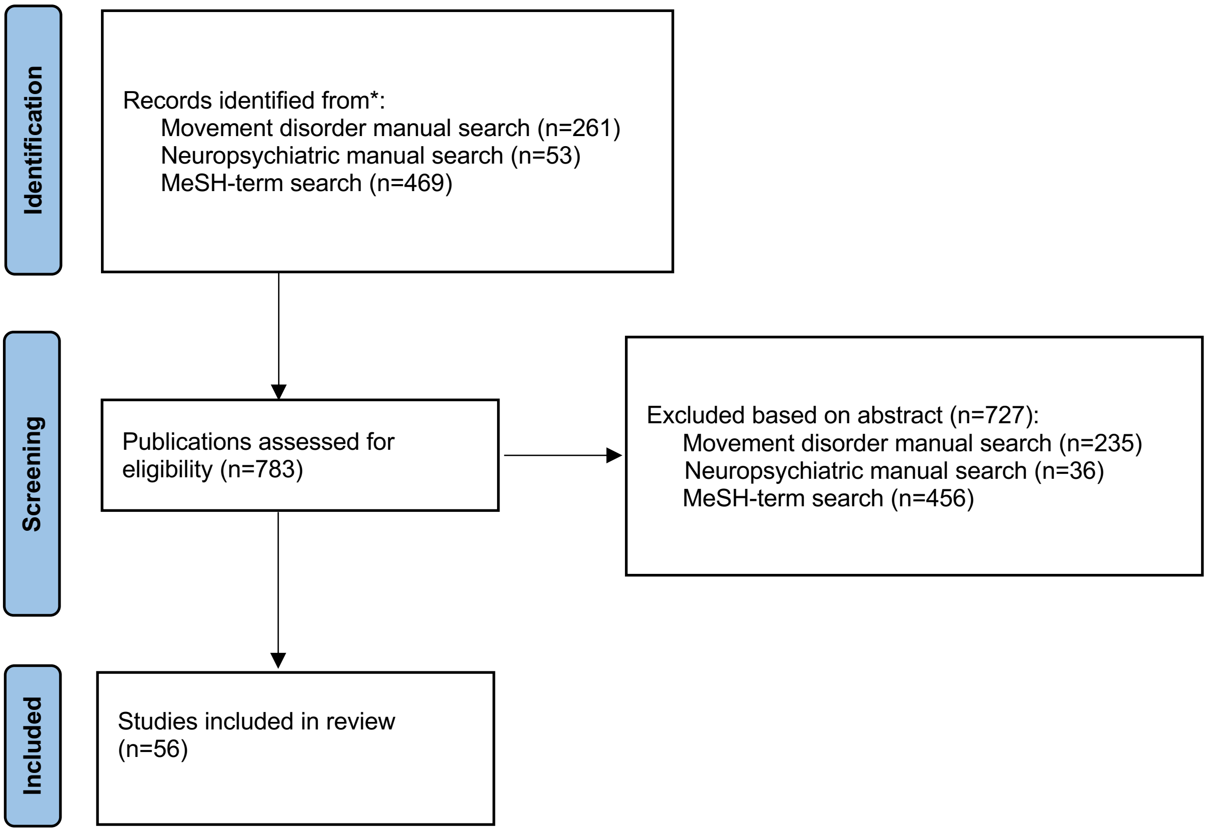

Supplemental Figure 2. PRISMA-style literature search flowchart. 783 publications were identified from three unique components of the literature search. 727 publications were excluded based on review of abstracts, resulting in the 56 unique papers reported here.

Supplementary Information

Metrics for evaluating machine learning model

**Metric**	**Formula**	**Description**
Accuracy	$\frac{True\ Positives + True\ Negatives}{True\ Positives + True\ Negatives + False\ Positives + False\ Negatives}$	Assesses how well the model makes the correct predictions. This metric could be misleading in, for example, unbalanced datasets. If a dataset had 1000 total data points split amongst 900 patients with PD and 100 healthy controls, a model that simply classifies every patient as having PD would have an accuracy of 900/1000 or 90%.
Area Under the Curve (AUC)	Area under the receiver operator curve (ROC), which plots false positive rate (1-specificity) on the x-axis and true positive rate (sensitivity) on the y-axis.	Assesses how well a model distinguishes between classes. An AUC of 0.5 indicates the model has no discriminatory ability. The interpretation of what a 'good' AUC is will depend on that specific model's application.<br><br>However, in general, an AUC between 0.7 and 0.8 is considered acceptable, 0.8 to 0.9 is considered excellent, and > 0.9 is considered outstanding[1].
Sensitivity	$\frac{True\ Positives}{True\ Positives + False\ Negatives}$	Assesses how well the model finds true positives. The model from the "Accuracy" row's description would have a sensitivity of 900/(900 + 0) or 100% because it is adept at finding true positives (i.e., classifying patients as PD when they do in fact have PD).
Specificity	$\frac{True\ Negatives}{True\ Negatives + False\ Positives}$	Assesses how well the model finds true negatives. Penalizes models for "sloppy" classifications (e.g., indiscriminately classifying everything in one class). The model from the "Accuracy" row's description would have a sensitivity of 0/(0 + 100) or 0% because it never correctly marked a healthy control (negative) classification. The 100 in the denominator corresponds to the number of false positives (i.e., number of times the model classified a HC as a patient with PD).
F1 score	$2\ x\ \frac{Precision\ x\ Recall}{Precision + \ Recall}$	Addresses the how well the model balances the precision/recall tradeoff inherent to classification tasks. The F1 score is the harmonic mean of precision (also known as positive predictive value; TP/TP + FP) and recall (also known as sensitivity; TP/TP + FN), therefore penalizing extreme values in a way that the arithmetic mean cannot.

Non-neural networks

Technique	Supervised / Unsupervised	Description
K-nearest neighbor (KNN)	Supervised	KNN is a classification algorithm that attempts to assign a data point into existing categories of data. For example, KNN can be applied to accelerometer data to determine if an individual aligns more with patients with PD or control patients. The first step of KNN involves clustering the data. This can be accomplished through multiple clustering algorithms including, but not limited to, principal components analysis (PCA) and k-means clustering. Once the data is clustered, KNN can take a new data point and classify it into an existing cluster based on the new data point's Euclidian distance from the existing clusters. This step depends on a parameter called the "K" value. The "K" value determines how many existing data points will be used when classifying a new data point. For example, if K equals 3, the algorithm would classify the new data point based on its 3 nearest data points. If, for example, 2 of those data points are in cluster A and the other is in cluster B, the new data point would be assigned to cluster A.
Support Vector Machine (SVM)	Supervised	SVM is a binary classification algorithm that attempts to find a line, plane, or hyperplane that can segment data into 2 categories. Implementing SVMs typically requires use of a kernel function. In essence, a kernel function is a data transformation that attempts to take data in one dimension and project it into another dimension to better create separation between categories within the data. For example, a polynomial kernel function might take one-dimensional data and square it, thereby projecting the data into two dimensions, before finding a line that can separate the data into two categories. Data with more complicated decision boundaries between the categories can be analyzed using more sophisticated kernel functions, such as the radial basis function.
Naïve-Bayes	Supervised	Naïve-Bayes is a Bayes Theorem-based classification algorithm. Bayes Theorem states that: $P(A\|B) = \frac{P(B\|A)*P(A)}{P(B)}$. Using this theorem, this classification algorithm can calculate the probability that a new data point belongs to a certain category (e.g., PD patient, non-PD patient), given a certain set of parameters are true (e.g., festinating gait is present, pill-rolling tremor is present).
Logistic regression	Supervised	Logistic regression is a classification algorithm that utilizes a sigmoid function to create non-linear decision boundaries between different classes in a dataset. One of the most salient features of the sigmoid function is its ability to generate a probability, between 0 and 1, that a data point belongs to a certain data class.
Decision trees	Supervised	Decision trees use features in the training data set to create a series of yes/no questions that can classify a new data point. The decision tree algorithm cycles through all combinations of questions that can be asked using the features in a data set (e.g., Does the patient exhibit festinating gait?, Is the patient > 50 years old), to ultimately determine which questions best discriminate data points into their respective categories. Decision trees can be combined with a technique called "bootstrap aggregating" or "bagging", resulting in bagged decision trees. Bagging trains multiple decision trees, each time using a subset of the training data, and decides on the final classification based on a majority vote of the trees, thereby lowering model variance and increasing accuracy.
Random forest	Supervised	Random forest is a classification algorithm that is a variation of bagged decision trees. Random forest uses a majority vote technique similar to that of bagged decision trees to classify data. However, unlike bagged decision trees, random forest trees are created using not just a subset of the training data, but also a subset of the features within the data, thereby further lowering the variance of the algorithm.

Neural networks

**Technique**	**Supervised / Unsupervised**	**Description**
Neural network (traditional)	Supervised	Neural networks can be used for multiple purposes, including data classification. Neural networks can be broken down into three components: input layer, hidden layer(s), and output layer. The input layer consists of nodes corresponding to the features within a data set. For example, if each sample in a data set had 3 features (e.g., patient age, weight, presence (y/n) of pill-rolling tremor), the input layers would have 3 nodes. The number of hidden layers and nodes within each layer can be optimized by trial and error in order to maximize the network's performance. In the case of binary classification, the output later would have 2 nodes, corresponding to the 2 possible classifications. Each node is connected to every node in the next layer by a scaling factor (weight) and an addition (bias). The output of this calculation is then fed into an activation function (e.g., rectified linear unit, sigmoid function) in order to introduce non-linearities into the network. The outputs from the activation function of all the nodes in one layer are summed and fed into the corresponding node in the next layer. The weights and biases in the network are initially randomized. Using minimization algorithms like gradient descent, a technique called backpropagation adjusts the weights and biases until the error between the training data set and the network's prediction is minimized. The network's training is then complete and can be assessed using the test data set.
Convolutional neural network (CNN)	Supervised	Convolutional neural networks are used to analyze images. The input layer is a 3 dimensional matrix that corresponds to number of rows, columns, and colors - typically 1 for a black and white image or 3 (red, green, and blue) for a color image. There are 2 main types of layers in the remaining CNN: convolutional layers and pooling layers. Convolutional layers convolve a 2 dimensional filter with the entire image, thereby reducing the size of the image. Convolutional layers mathematically extract specific features from the image (e.g., edges) depending on the weights in the filter, which are optimized using backpropagation in a similar fashion to traditional neural networks. After convolution, the data go through pooling layers in order to down sample the data by, for example, taking the maximum value of a group of adjacent cells in the image. At the end of the network, the image data is flattened into one column and used as an input into a fully-connected layer, after which there is an output layer used for classification.
